# Preparation of Self-Coating Al_2_O_3_ Bonded SiAlON Porous Ceramics Using Aluminum Dross and Silicon Solid Waste under Ambient Air Atmosphere

**DOI:** 10.3390/ma16165679

**Published:** 2023-08-18

**Authors:** Zhaoyang Liu, Junyang Wang, Zixu Zhao, Qiuyu Yang, Lihang Qin, Kaichen Zhang, Xiangnan Wang, Nan Su, Tianpeng Wen, Lei Yuan, Jingkun Yu

**Affiliations:** 1 Key Laboratory for Ecological Metallurgy of Multimetallic Ores (Ministry of Education), Northeastern University, Shenyang 110819, China; 2 School of Metallurgy, Northeastern University, Shenyang 110819, China; 3 School of Materials Science and Engineering, Shandong University of Science and Technology, Qingdao 266590, China; 4 Sinosteel Equipment & Engineering Co., Ltd., Beijing 100080, China

**Keywords:** aluminum dross, silicon solid waste, SiAlON, refractory

## Abstract

Al_2_O_3_-bonded SiAlON ceramic with self-coating was prepared using aluminum dross and silicon solid waste as starting materials under ambient air conditions. The changes in phase, microstructure, and physical properties of the ceramic with temperature were analyzed and the formation mechanism of the SiAlON phase was elucidated. The results showed that higher temperature was more suitable for the preparation of SiAlON ceramics. As the temperature increased from 1400 to 1600 °C, the main phases in the ceramic transformed from mullite, Al_2_O_3_, and SiAlON to Al_2_O_3_ and SiAlON. An Al_2_O_3_-rich layer spontaneously coated the surface of the porous ceramic as Al melted and oxidized at high temperature. The thickness of this layer decreased as the temperature increased. The presence of Al_2_O_3_-rich coating layer impeded air flow, allowing nitriding of Si and Al, and the formation of the SiAlON phase in ambient air conditions. This study not only presents a strategy to successfully recycle aluminum dross and silicon solid waste but also offers a straightforward approach to preparing SiAlON material in air atmosphere.

## 1. Introduction

SiAlON is a solid solution that combines the benefits of Si_3_N_4_ and Al_2_O_3_ [1,2]. It is generally formed by mixing Si_3_N_4_ with AlN and Al_2_O_3_, resulting in a material with excellent mechanical strength, low thermal expansion coefficient, exceptional wear resistance, and chemical corrosion resistance [3,4,5]. It is widely used in industries such as metallurgy, electronics, chemical, and aerospace as a high-temperature structural ceramic [6,7,8,9]. Several methods are available for synthesizing SiAlON materials, including direct synthesis (high-temperature solid reaction), self-propagating high-temperature synthesis, and reduction nitriding process [10,11,12]. For example, Guo et al. [13], using α-Si_3_N_4_, AlN, and Al_2_O_3_ as the raw materials, synthesized SiAlON ceramics by spark plasma sintering at 1600–1750 °C under vacuum atmosphere. Li et al. [14] fabricated β-SiAlON/hexagonal boron nitride (h-BN) nanocomposite by a precursor infiltration and pyrolysis (PIP) route using Si_3_N_4_, Al_2_O_3_, and AlN as the starting materials. Tian et al. [15] fabricated β-SiAlON powders under nitrogen atmosphere using Al, Si, α-Al_2_O_3_, and trace Y_2_O_3_ powders as starting materials, and studied the effect of Y_2_O_3_ powder on the oxidation resistance of β-SiAlON. The results indicated that at the final oxidation period, denser glass film formed on the surface of β-SiAlON with the incorporation of Y_2_O_3_, which reduced the opportunity for O_2_ contact with β-SiAlON, thus decreasing the oxidation rate. Due to the sensitivity of SiAlON ceramics to oxygen at high temperatures, the above methods often require expensive nitrides (Si_3_N_4_ and AlN) as raw materials and need to be performed under high purity nitrogen or vacuum atmosphere. Additionally, the reaction temperature is usually relatively high, even up to 1750 °C, making the preparation process complex, expensive, and unsuitable for large-scale applications [16,17]. Thus, finding a more economical and efficient method for preparing SiAlON ceramics has become imperative.

The rapid growth of solid wastes poses a significant challenge that has necessitated the development of clean technologies for their appropriate management. The high volume of aluminum production has led to a large amount of solid waste in the form of aluminum dross, resulting in a severe environmental concern [18]. Typically, the production of one ton of molten aluminum from aluminum ore and secondary aluminum resources generates 15–25 kg and 80–150 kg of aluminum dross, respectively. Thus, millions of tons of dross are generated yearly, most of which are harmfully disposed of in landfills globally [19,20]. The accumulation of aluminum dross not only leads to resource wastage but also creates serious public safety and environmental problems. With the rise in environmental awareness, researchers are making great efforts to address this issue, with more focus on recycling aluminum dross [21]. Several aluminum dross treatment methods are available, with the majority targeting the recovery of aluminum and alumina. For example, heating aluminum dross in a rotating furnace utilizing salt flux can separate molten Al from the solid oxide fraction while protecting the Al metal against oxidation [22]. Sarker et al. [23] extracted alumina from aluminum dross by an acid dissolution process, and studied the effects of various parameters, e.g., temperature, acid concentration, and leaching time, on the extraction of alumina to optimize the dissolution process. However, recycling aluminum dross poses significant challenges due to its diverse composition and similar properties. Moreover, the current recycling methods generate a large quantity of water-leachable salts and compounds, which contaminate the environment when released into landfills [24]. Different from the concept of separation and purification, some researchers have utilized aluminum dross directly to prepare related refractories. For example, Su et al. [25] synthesized Al_2_O_3_-SiO_2_-rich castables for reheating furnaces through progressive replacement of clay by aluminum dross. Zhang et al. [26] reutilized secondary aluminum dross for preparation of MgAl_2_O_4_, and studied the effect of rare earth oxides on the densification behavior of MgAl_2_O_4_. Considering that aluminum dross primarily consists of Al, Al_2_O_3_, and AlN, it is suitable for the production of SiAlON ceramic by the addition of the appropriate amount of silicon. Moreover, this can be achieved without any separation treatment for aluminum dross. Coincidentally, massive amounts of silicon solid waste are generated in the process of crystal silicon cutting during the development of the photovoltaic industry [27,28]. Researchers have focused on the effective recovery of high purity Si from silicon solid wastes for the sustainable development of photovoltaic industry, and various methods have been developed [29,30,31,32]. In fact, these silicon wastes have small particle sizes and high activity, thereby serving as ideal silicon sources to manufacture SiAlON material.

To the best of our knowledge, there are currently no reports on the preparation of SiAlON ceramics using aluminum dross and silicon waste, especially in air atmosphere. This is because both silicon nitride and aluminum nitride are prone to oxidation reactions in air atmosphere at high temperature, making it difficult to form the SiAlON phase. Previous studies on the synthesis of the SiAlON phase were mostly conducted under high-purity nitrogen gas. In this study, the unique characteristics of aluminum dross and silicon solid waste are utilized to form a coating layer during the high temperature reaction process, providing conditions for the formation of the SiAlON phase inside the sample. The study analyzed the phase composition, microstructure, and physical properties of the materials synthesized utilizing aluminum dross and silicon solid waste at different temperatures, and they also clarified the process of SiAlON phase formation. The findings of this study suggest the potential for using these two types of solid wastes for synthesizing SiAlON material, even in the absence of a pure nitrogen atmosphere.

## 2. Materials and Methods

### 2.1. Raw Materials

**Figure 1:**
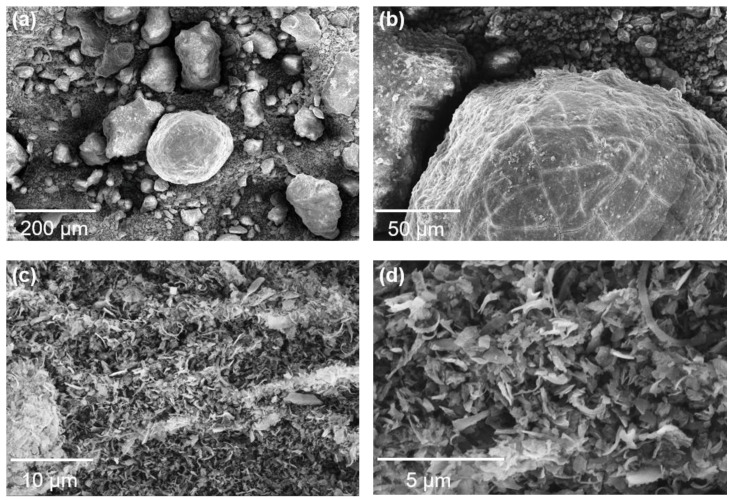
SEM images of (**a**,**b**) aluminum ash and (**c**,**d**) silicon solid waste.

**Figure 2:**
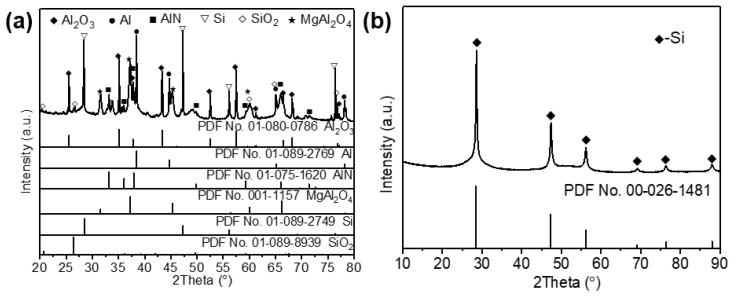
XRD patterns of (**a**) aluminum ash and (**b**) silicon solid waste.

**Figure 3:**
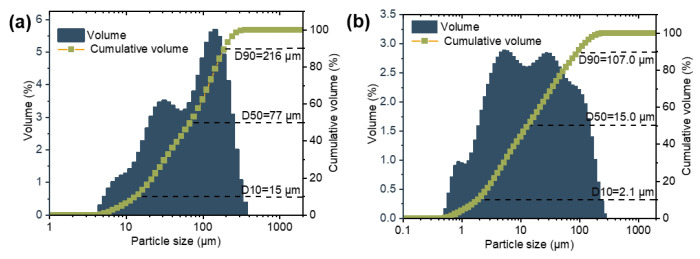
Particle size distributions of (**a**) aluminum ash and (**b**) silicon solid waste.

Aluminum dross and silicon solid waste were used as starting materials to produce sintered samples with desirable properties. The elemental contents of aluminum dross were determined by X-ray fluorescence (XRF) and presented in Table 1. The silicon solid waste comprised approximately 99% silicon according to XRF analysis. Figure 1 presents the SEM images of the aluminum dross and silicon solid waste. It is evident from Figure 1a,b that the aluminum dross consists of particles with varied sizes and distinct morphologies, suggesting the presence of multiple phases. SEM images of the silicon solid waste in Figure 1c,d show that it mainly consists of small fragments. While most sizes are relatively uniform, there are also some large agglomerates present. Results of XRD analysis (Figure 2a) reveal that the main phases in the aluminum dross include Al, Al_2_O_3_, AlN, and Si. Therefore, as shown in Table 1, the aluminum element originates from three substances: Al, Al_2_O_3_, and AlN. Quantitative testing results reveal that the mass fractions of these three components are as follows: Al (33.60%), Al_2_O_3_ (43.23%), and AlN (6.56%). Figure 2b shows that the primary component of silicon waste is silicon element, with very few impurities, which is consistent with the XRF results. The particle size distribution shown in Figure 3 indicates that D50 values for aluminum dross and silicon solid waste are 77 and 15 μm, respectively.

### 2.2. Preparation of Al_2_O_3_-Bonded SiAlON Ceramic

**Figure 4:**
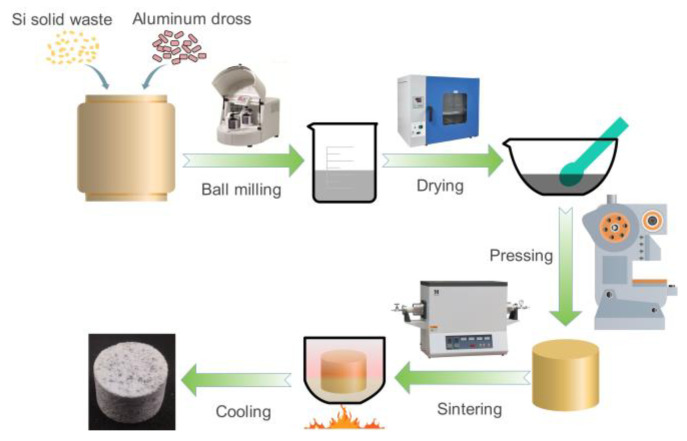
Illustration of the preparation process of Al_2_O_3_-bonded SiAlON ceramic with self-coating.

Aluminum dross and silicon solid waste were added into a ball milling jar, and appropriate amounts of agate balls and deionized water were added simultaneously. A planetary ball mill was used to uniformly mix the raw materials for 1 h. Then, the slurry was transferred to a drying oven and dried for 24 h. The dried powder mixture was placed into a cylindrical mold, and compacted using an automatic hydraulic press at a rate of 0.5 MPa/s. The molding pressure was maintained at 200 MPa for 10 min. Subsequently, pressure release and demolding treatments were performed to obtain green bodies with dimensions of Φ15 × 15 mm^3^. The green bodies were placed in a corundum crucible with a lid. The corundum crucible with the sample was then placed into a tube resistance furnace and the temperature was raised at a rate of 5 °C·min^−1^ to 1400, 1500, and 1600 °C, respectively, and the set temperature was maintained for 3 h. After cooling to room temperature, the ceramic specimen was cut radially and one part was set with epoxy resin. After the resin was solidified, the specimen with resin was ground and polished using standard procedures. Another part of the sample was broken off directly for cross-section observation. The illustration of the preparation process of Al_2_O_3_-bonded SiAlON ceramic with self-coating can be seen in Figure 4.

### 2.3. Characterization

The apparent porosity and bulk density of the sintered samples were evaluated according to the Archimedes principle, and the calculation method can be seen in previous work [33]. Cold compressive strengths of the sintered samples were measured by using an electronic material testing machine (WOW-100, Chuangbai Equipment Co., Ltd.; Jinan, China) at a load rate of 0.5 mm·min^−1^. The cold compressive strength was calculated as the applied force divided by the area of cross section. A laser particle analyzer (Mastersizer 3000, Malvern, UK) was employed to determine the particle size distribution of the raw materials. Crystallographic phases of the raw materials and sintered samples were identified via X-ray diffraction (XRD) with copper Kα radiation (λ = 0.154056 nm) under an accelerating voltage of 40 kV. XRD was performed over a 2*θ* range of 10° to 80° at a scanning rate of 5°·min^−1^ at room temperature. Additionally, the microstructure and morphology of the raw materials as well as the cross section and polished surface of ceramic specimens were observed using scanning electron microscope (SEM, S4800, Tokyo, Japan). The elemental composition of specific areas was determined by energy-dispersive X-ray spectroscopy (EDS).

## 3. Results and Discussion

**Figure 5:**
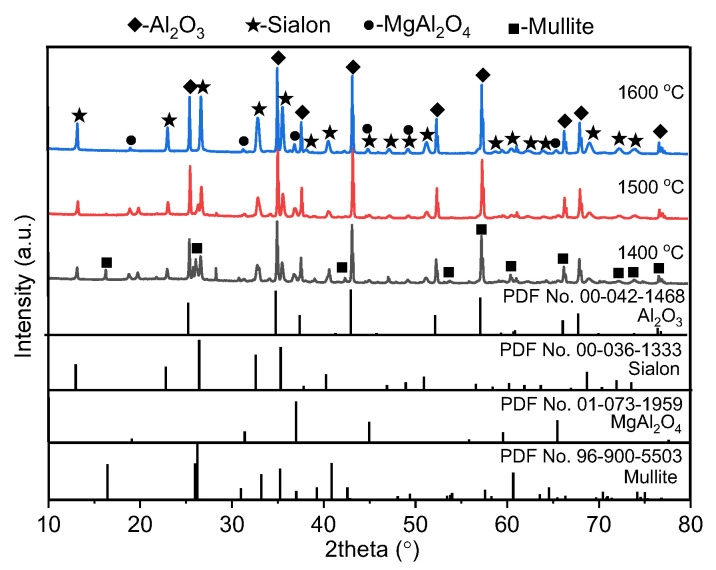
XRD patterns of Al_2_O_3_-bonded SiAlON porous ceramics with self-coating.

The XRD patterns of the Al_2_O_3_-bonded SiAlON porous ceramics with self-coating obtained at different temperatures are presented in Figure 5. At 1400 °C, the main phases observed were Al_2_O_3_, mullite, and SiAlON, with minor amounts of MgAl_2_O_4_ phase. However, the Al and Si phases in the starting material disappeared, indicating that all metal Al and Si underwent both nitriding and oxidation to form mullite and SiAlON after sintering. Upon increasing the temperature to 1500 °C, the diffraction peak corresponding to mullite disappeared, while the intensities of the diffraction peaks for Al_2_O_3_ and SiAlON increased. The disappearance of mullite phase indicated that the increase in temperature suppressed the oxidation of metallic silicon and promoted its nitridation. Leary et al. indicated that the aluminosilicate might form both crystalline and amorphous intermediates [34]. The amorphous phase then separates into Al-rich and Si-rich phases; the former converts via X-SiAlON to higher z-value β-SiAlON, whereas the latter converts via O′-sialon to β-SiAlON of low z-value. With a further increase in temperature to 1600 °C, the intensities of diffraction peaks for Al_2_O_3_ and SiAlON further increased, while the intensity of the diffraction peak associated with MgAl_2_O_4_ phase remained relatively constant. The XRD results indicated that the SiAlON phase was more likely to be formed at higher temperatures. Tu et al. also indicated that a higher firing temperature is beneficial for the solid solution of Si_3_N_4_ and Al_2_O_3_ to transform into SiAlON [35]. 

**Figure 6:**
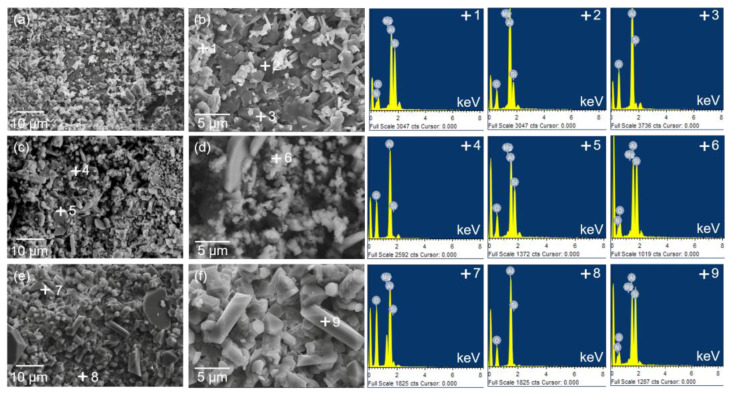
SEM images and EDS results of Al_2_O_3_-bonded SiAlON porous ceramics after sintering at different temperatures: (**a**,**b**) 1400 °C, (**c**,**d**) 1500 °C, (**e**,**f**) 1600 °C.

Figure 6 displays the SEM images of the fracture surface of Al_2_O_3_-bonded SiAlON porous ceramics, along with the corresponding EDS results for the selected points presented in Table 2. At a temperature of 1400 °C, the light gray phase was rich in Si, Al, O, and N elements, indicating that it was composed SiAlON. The coarser phases with dark grey were mullite (+2) and Al_2_O_3_ (+3), according to the composition and proportion of the elements. With the increase in temperature to 1500 °C, the sample primarily consisted of granular SiAlON (+6) with an average diameter of approximately 2 μm. Flaky alumina (+5) and a few mullite particles (+4) were also observed. Upon reaching a temperature of 1600 °C, the main phases remained unchanged, but the mullite phase disappeared. The sizes of granular SiAlON and flaky alumina were noticeably increased. This may be attributed to the increase in temperature, which accelerates the mass transport and Ostwald ripening process, similar to the preparation of Si_3_N_4_ [36]. A small amount of MaAl_2_O_4_ phase was also observed in the sample prepared at 1600 °C. The SEM and EDS results for the phase evolution with temperature were found to be consistent with the XRD results displayed in Figure 5. 

**Figure 7:**
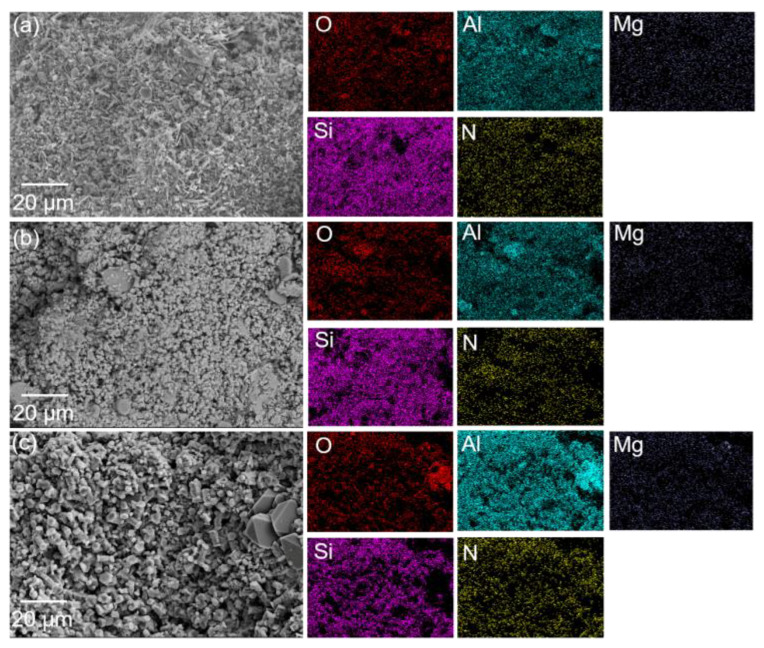
SEM images and EDS-mapping results of Al_2_O_3_-bonded SiAlON porous ceramics after sintering at different temperatures: (**a**) 1400 °C, (**b**) 1500 °C, (**c**) 1600 °C.

In order to better observe the distribution of different phases in the samples, EDS mapping was performed on the samples at three different temperatures. Figure 7 shows the distribution of the elements in the microstructure of the Al_2_O_3_-bonded SiAlON porous ceramics. The mapping results suggested that the four main elements in the samples, namely, O, N, Si, and Al, were distributed relatively evenly, indicating a uniform distribution of the SiAlON and alumina phases. Additionally, a small amount of Mg element was present in the samples, mainly attributed to the MgAl_2_O_4_ phase, according to XRD results. As the temperature increased, the presence of larger alumina particles became more apparent. 

**Figure 8:**
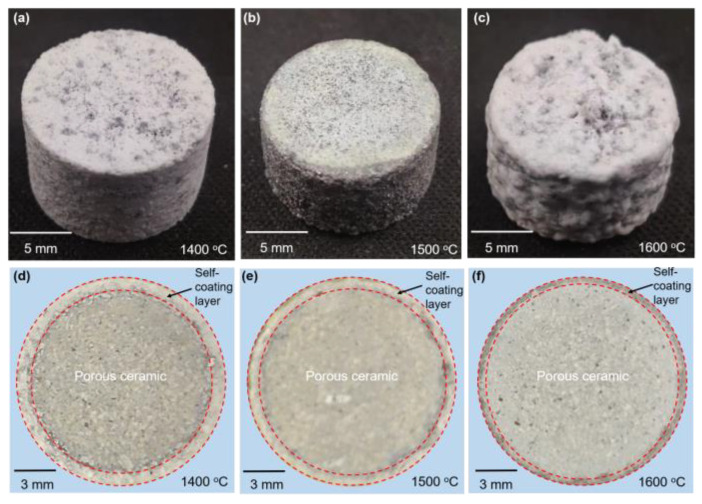
(**a**–**c**) Macroscopic views and (**d**–**f**) cross-sections of polished samples at different temperatures.

Figure 8 shows the exterior and cross-section views of the samples at three temperature conditions. It can be observed that a white Al_2_O_3_-rich coating was present on the surface of the sample at the macroscopic level. With the increase in temperature from 1400 to 1600 °C, the thickness of the coating layer decreased from 0.63 to 0.41 mm. The main reason for the reduction in the thickness of the coating was that aluminum is more prone to react with oxygen at higher temperatures, leading to the rapid formation of a dense layer of Al_2_O_3_ that coats the surface of the sample. This makes subsequent oxidation reactions of the aluminum more difficult but promotes the nitridation reaction. Therefore, the thickness of the coating decreased with the increase in temperature.

**Figure 9:**
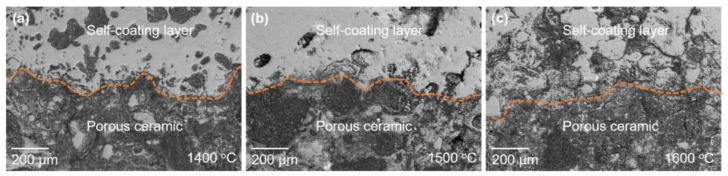
SEM images of the interface between coating and Al_2_O_3_-bonded SiAlON porous ceramics.

Figure 9 shows the SEM images of interfaces between coating layers and Al_2_O_3_-bonded SiAlON porous ceramics. Due to the treatment of epoxy resin embedding before polishing and SEM observation, some liquid resin infiltrated into the interior of the porous ceramic. As shown in Figure 9, the gray-white portion represents the ceramic phase, while the black portion represents the pores filled with resin. There were significantly fewer pores in the coating layer compared to the interior of the ceramic sample. Although the coating layer also contained a few pores, it still formed a continuous structure, effectively impeding the diffusion of external air into the interior of samples [37,38]. Therefore, the oxidation of SiAlON was greatly inhibited. Zhao et al. also effectively improved the oxidation resistance of the SiAlON ceramics by adding h-BN to the raw material to form a borosilicate glass phase on the surface of the SiAlON ceramics [39].

**Figure 10:**
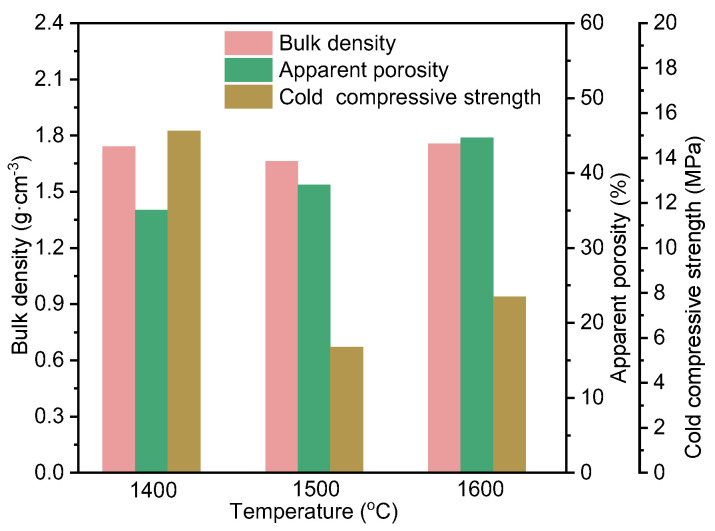
Bulk density, apparent porosity, and cold compressive strength of self-coating Al_2_O_3_-bonded SiAlON porous ceramics.

Figure 10 shows the results of bulk density, apparent porosity, and cold compressive strength of the Al_2_O_3_-bonded SiAlON porous ceramics with self-coating layer. As seen from the figure, the bulk density remained nearly unchanged at around 1.75 g·cm^−3^ as the temperature increased. However, the apparent porosity increased gradually with the increase in temperature. This was mainly due to the fact that the specimens contained more SiAlON at higher temperatures, which caused them to expand and form some microcracks, resulting in increased porosity. Moreover, the self-coating layer became thinner as the temperature increased, leading to a higher overall porosity of the specimens. The cold compressive strength decreased and then slightly increased with the increase in temperature from 1400 to 1600 °C. Multiple factors influence the cold compressive strength, such as the phase composition, microstructure, and porosity of the samples [40,41,42]. Due to the increased porosity of the sample with the increase in temperature, the cold compressive strength decreased significantly. Moreover, the decomposition of mullite may also cause the decrease in strength [43]. However, the cold compressive strength at 1600 °C was slightly higher than that at 1500 °C. One possible reason was that the coating layer at 1600 °C showed signs of melting, resulting in a higher strength after cooling and solidification, thereby increasing the overall strength of the sample.

Many studies have reported the formation of SiAlON whiskers using Si and Al–based compounds as starting materials under nitrogen atmosphere, and the vapor–solid (VS) and used vapor–liquid–solid (VLS) growth mechanisms to explain the growth of SiAlON whiskers [44,45]. The composition of synthesis gas (containing SiO and Al_2_O) makes it an important intermediate phase, which significantly influences the preparation and growth mechanism of the whiskers [46]. However, in this study, uniform granules were found in the porous samples instead of whiskers, which may indicate that the gas phase was less involved during the growth process of the SiAlON phase. The fabrication process of Al_2_O_3_-bonded SiAlON porous ceramics with a self-coating layer can be described as follows: under crucible sealing conditions, the metal Al in the dross melts and diffuses towards the surface of sample as the temperature increases. Upon encountering oxygen in the atmosphere, the Al is oxidized, resulting in the formation of an Al_2_O_3_-rich coating layer (Equation (1)). This layer acts as a barrier, preventing air diffusion into the inner part of the specimen and creating favorable conditions for the nitridation of Si and Al. Consequently, Si in the solid waste undergoes oxidation and nitridation reactions, leading to the production of SiO_2_ and Si_3_N_4_ (Equations (2)–(5)). It is widely considered that the formation of Si_3_N_4_ is the key for synthesizing SiAlON [47]. Similarly, Al undergoes reactions that produce Al_2_O_3_ and AlN (Equations (1) and (6)). The reaction between SiO_2_ and Al_2_O_3_ forms mullite (Equation (7)), while the reaction among Si_3_N_4_, AlN, and Al_2_O_3_ leads to the formation of the SiAlON phase (Equation (8)). Due to the existence of self-coating layer, the SiAlON phase is stable at high temperature, even though the oxidation of SiAlON can occur above 700 °C in normal cases [48]. Additionally, a small amount of MgO present in the aluminum dross reacts with Al_2_O_3_ to produce MgAl_2_O_4_ (Equation (9)). At temperatures of 1500 and 1600 °C, the Al_2_O_3_-rich coating layer rapidly forms, facilitating the formation of SiAlON phase. Consequently, there is an increase in the content of the SiAlON phase and nearly no mullite phase in the sample:

4 Al (s, l) + 3 O_2_ (g) → 2 Al_2_O_3_ (s) (1)

Si (s, l) + O_2_(g) → SiO_2_ (s) (2)

3 Si (s, l) + 2 N_2_ (g) → Si_3_N_4_ (s) (3)

Si (s) + SiO_2_ (s) → 2 SiO (g) (4)

6 SiO (g) + 4 N_2_ (s) → 2 Si_3_N_4_ (s) + 3 O_2_ (g) (5)

2 Al (s, l) + N_2_ (g) → 2 AlN (s) (6)

3 Al_2_O_3_ (s) + 2 SiO_2_ (s) → 3 Al_2_O_3_·2SiO_2_ (s) (7)

4 Si_3_N_4_ (s) + 2 AlN (s) + 2 Al_2_O_3_ (s) → 3 Si_4_Al_2_O_2_N_6_ (s) (8)

MgO (s) + Al_2_O_3_ (s) → MgAl_2_O_4_ (s) (9)

## 4. Conclusions

Porous ceramics consisting of Al_2_O_3_-bonded SiAlON were successfully prepared using aluminum dross and solid silicon waste as starting materials under ambient air conditions. The phase compositions, microstructure, and physical properties of the ceramics were investigated. Additionally, the formation mechanism of the SiAlON phase was also revealed. Based on the obtained results, the main conclusions can be summarized as follows:

1. Higher temperature was more suitable for the formation of SiAlON phase. With the increase in temperature from 1400 to 1600 °C, the mullite phase in the Al_2_O_3_-bonded SiAlON porous ceramic disappeared, and the content of Al_2_O_3_ and SiAlON phases increased.
2. A layer of Al_2_O_3_-rich coating spontaneously formed on the surface of the Al_2_O_3_-bonded SiAlON porous ceramic, which decreased in thickness with increase in temperature. The formation of the Al_2_O_3_-rich coating layer and the sealing of crucible obstructed the air flow, facilitating the nitriding of Si and Al and the formation of SiAlON phase under ambient air conditions.
3. The apparent porosity of the Al_2_O_3_-bonded SiAlON porous ceramic increased with the increase in temperature, while the bulk density and cold compressive strength decreased first and then increased with the increase in temperature. These changes were mainly related to the phase evolution and the microstructure of Al_2_O_3_-bonded SiAlON porous ceramic and Al_2_O_3_-rich coating layer.

## Figures and Tables

**Table 1:** XRF results of aluminum dross (wt%).

	Al	Si	Mg	Ca	K	Ti	Fe	Others
Contents	77.42	9.99	3.53	2.34	2.24	2.20	0.91	1.37

**Table 2:** EDS results of the selected spots (at%).

	Si	Al	O	N	Mg	Possible Phase
+1	19.25	19.17	30.19	30.36	1.02	Sialon
+2	15.66	42.22	41.15	-	0.97	Mullite
+3	3.31	37.09	59.60	-	-	Al_2_O_3_
+4	24.09	27.29	46.95	-	1.67	Mullite
+5	1.59	33.11	65.29	-	-	Al_2_O_3_
+6	20.33	14.83	31.71	32.17	0.96	Sialon
+7	1.90	24.40	64.02	-	9.67	MgAl_2_O_4_
+8	20.37	14.71	28.44	35.41	1.06	Sialon
+9	2.08	43.20	54.71	-	-	Al_2_O_3_

## Data Availability

Not applicable.
